# Performance of quantitative measurements in [^18^F]fluorocholine positron emission tomography/computed tomography for parathyroid imaging (P2TH study)

**DOI:** 10.3389/fmed.2022.956580

**Published:** 2022-08-02

**Authors:** Nicolas Jacquet-Francillon, Isabelle Morelec, Natacha Germain, Jean-Michel Prades, Vincent Habouzit, Christophe Mariat, Pierre-Benoit Bonnefoy, Nathalie Prevot

**Affiliations:** ^1^Department of Nuclear Medicine, Saint-Étienne University Hospital, University of Saint-Étienne, Saint-Étienne, France; ^2^Department of Nuclear Medicine, Hospices Civils de Lyon, Lyon, France; ^3^Division of Endocrinology, Diabetes, Metabolism and Eating Disorders, Centre Hospitalo-Universitaire (CHU) de Saint-Étienne, Saint-Étienne, France; ^4^Eating Disorders, Addictions and Extreme Bodyweight Research Group (TAPE) EA 7423, Université Jean Monnet, Saint-Étienne, France; ^5^Department of Otorhinolaryngology, Head and Neck Surgery, Hospital Saint-Étienne, Saint-Étienne, France; ^6^Laboratory of Human Anatomy, Faculty of Medicine, University of Saint-Étienne, Saint-Étienne, France; ^7^Department of Nephrology, Dialysis and Renal Transplantation, Hôpital Nord, Centre Hospitalo-Universitaire (CHU) de Saint-Étienne, Jean Monnet University, Université de Lyon, Saint-Étienne, France; ^8^Groupe Immunité des Muqueuses et Agents Pathogènes GIMAP, EA 3065, University of Jean Monnet and Université de Lyon, Saint-Étienne, France; ^9^INSERM, U1059, SAINBIOSE, Univ Lyon, Univ Saint-Etienne, Saint-Etienne, France

**Keywords:** SUV, [^18^F]fluorocholine, parathyroid, PET/CT, PTH, adenoma, hyperplasia, semi quantitative analysis

## Abstract

**Objective:**

[^18^F]Fluorocholine positron emission tomography/computed tomography (PET/CT) is used frequently in addition to [^99m^Tc]Tc-Sestamibi scintigraphy and ultrasonography for the location of hyperfunctioning parathyroid glands. The aim of this study is to evaluate the performance of quantitative criteria in [^18^F]fluorocholine PET/CT for localization of hyperfunctioning parathyroid glands. The secondary objective is to highlight a correlation between the detection rate of [^18^F]fluorocholine PET/CT and serum parathyroid hormone (PTH) level.

**Materials and methods:**

In two academic centers, we retrospectively included patients with biological hyperparathyroidism (HPT) and who had [^18^F]fluorocholine PET/CT. After a visual analysis, to measure the overall performance of [^18^F]fluorocholine PET/CT, a blind reading was carried out with standardized measurements of maximum standardized uptake value (SUV_max_), liver ratio, thyroid ratio, and size ratio. We analyzed the quantitative criteria of [^18^F]fluorocholine PET/CT compared to the histological results, in particular to identify differences between adenomas and hyperplasias. We compared the performance of each quantitative criterion to the overall sensitivity, specificity, positive predictive value (PPV), negative predictive value (NPV), and accuracy of [^18^F]fluorocholine PET/CT. The detection rate of hyperfunctioning parathyroid glands was calculated in subgroups of serum PTH level.

**Results:**

The quantitative criteria in [^18^F]fluorocholine PET/CT were measured for 120 patients (135 lesions). The areas under the receiver operating characteristic (ROC) curve representing SUV_max_ and liver ratio were significantly increased. The optimal cut-off values represented by the maximum Youden index was >4.12 for SUV_max_ and >27.4 for liver ratio. Beyond certain threshold values of SUV_max_ (>4.12) or liver ratio (>38.1), all the lesions were histologically proven adenomas. SUV_max_ and liver ratio were significantly higher for adenomas than for hyperplasias and differential diagnosis (*p* = 0.0085 and *p* = 0.0002). The positivity of [^18^F]fluorocholine PET/CT was correlated with PTH level. Detection rates were 55.56, 75.56, and 87.5%, respectively, for serum PTH < 70, 70 to 120, and >120 ng/ml.

**Conclusion:**

Semi-quantitative measurements (SUV_max_ and liver ratio) should be considered as additional tools in interpretation of [^18^F]fluorocholine PET/CT. These quantitative parameters have lower overall performance but higher specificity than overall visual analysis in identifying an adenoma. Above certain threshold values, all lesions are adenomas. [^18^F]fluorocholine PET/CT confirms excellent performance for the detection of hyperfunctional parathyroids. For serum PTH levels < 70 ng/ml, the detection rate of [^18^F]fluorocholine PET/CT is strongly decreased.

## Introduction

Hyperparathyroidism (HPT) is a very common endocrine disorder. It is characterized by hypercalcemia associated with an elevated or inappropriately normal parathyroid hormone (PTH) serum level. Three forms can be distinguished: primary (pHPT), secondary (sHPT), and tertiary hyperparathyroidism. pHPT is mostly caused by a single adenoma, more rarely by hyperplasia, in some cases by a parathyroid carcinoma, and quite frequently by a multiple-gland disease (15–20%) (1). pHPT is newly diagnosed in 27 per 100,000 patients in the world, and its incidence is significantly increased over the last decades (2). Less well-known is the incidence of secondary and tertiary hyperparathyroidism. A study on 10,000 patients from the 2000s demonstrated a prevalence rate of 5.5% and an incidence rate of parathyroidectomy of 528 per 100,000 (3).

The treatment of pHPT and tertiary hyperparathyroidism is mainly surgical, by parathyroidectomy. The location of the pathological parathyroid gland represents a major issue in guiding surgical management (4). There are two main surgical techniques: unilateral mini-invasive surgery (MIP) and bilateral neck exploration. The results of MIP compared to those of bilateral exploratory surgery show a reduction in post-operative pain, surgery time, and number of symptomatic post-operative hypocalcemia (5–7). The rate of conversion of MIP to bilateral cervicotomy is estimated at 8–15% (8). The rate of surgery failure is estimated at 2–5% (9). Therefore, an increasingly reliable pre-surgical localization is essential.

Several meta-analyses (10–12) showed [^18^F]fluorocholine positron emission tomography/computed tomography (PET/CT) performance better than optimal imaging combination, namely, [^99m^Tc]Tc-Sestamibi scintigraphy with ultrasonography, for localization of hyperfunctional parathyroid glands. [^18^F]fluorocholine PET/CT appears effective for <10 mm lesions (13), ectopic glands, and brown tumors (14). In addition, patient dosimetry is lower than with the scintigraphy-ultrasonography combination (15). However, use of [^18^F]fluorocholine PET/CT as first-line imaging is controversial (16–19). Scintigraphy and echography are still recommended in first intention by the European Association of Nuclear Medicine (EANM) (20).

The analysis of quantitative measurements in [^18^F]fluorocholine PET/CT is the subject of few studies and is always a secondary objective. Our hypothesis is that quantitative criteria could facilitate interpretation and could allow for better specificity of the examination with harmonization of practices. Among the quantitative criteria investigated, Treglia et al. and Araz et al. (10, 21) proposed the use of maximum standardized uptake value (SUV_max_) to differentiate adenomas from hyperplasias. Treglia et al. and Piccardo et al. (10, 22) proposed the use of SUV_max_ and lesion-to-neck background ratio to identify hyperfunctional glands. Treglia et al. (10) had also investigated lesion-to-thyroid ratio to identify hyperfunctional glands. Piccardo et al. (22) described a correlation between SUV ratio to neck background and Ki67 expression in 18F-Choline PET/4D CT. Recently, Liberini et al. (23) compared histological features of parathyroid adenomas with [^18^F]fluorocholine positron emission tomography/magnetic resonance imaging (PET/MRI) uptakes. To our knowledge no study investigating [^18^F]fluorocholine PET/CT quantitative criteria in hyperparathyroidism in comparison to anatomopathological findings as main objective.

The aim of this study is to evaluate the performance of quantitative criteria in [^18^F]fluorocholine PET/CT for localization of hyperfunctioning parathyroid glands. The secondary objectives of this study are (i) to investigate the diagnostic efficacy of [^18^F]fluorocholine PET/CT depending on the level of PTH and (ii) to demonstrate a significant difference and cut-off between adenomas and hyperplasias regarding the defined quantitative criteria.

## Materials and methods

### Design and population

This study is a retrospective analysis of [^18^F]fluorocholine PET/CT realized in the nuclear medicine department of two centers (*Hôpital Nord, CHU de Saint-Etienne*, and *Hôpital de Lyon Sud, Hospices Civils de Lyon*) between December 2014 and December 2020.

Inclusion criteria were (i) patients aged ≥18 years with biologically proved primary, secondary, or tertiary hyperparathyroidism, (ii) negative or discordant [^99m^Tc]Tc-Sestamibi SPECT/CT scintigraphy and ultrasonography, (iii) [^18^F]fluorocholine PET/CT carried out in one of the two investigation centers, and (iv) histological results or at least 1 year of follow-up if surveillance decision. Exclusion criteria were as follows: (i) histological analysis carried out outside the two investigation centers; (ii) patient lost to follow-up; (iii) refusal or poor benefit risk ratio of the surgery; (iv) liver outside the scope of acquisition. All procedures are in accordance with the ethical principles of the institutions and with the Declaration of Helsinki of 1964. The study protocol was approved by a French ethics committee for research on nuclear medicine (record number: CEMEN2021-06). All the patients received an information letter about the creation of the study, the objectives, and their right of refusal.

### Surgery, histopathology, and intraoperative parathyroid hormone measurement

The decision to operate or to follow the patient without surgery was a multidisciplinary decision. Surgical criteria of the Fourth International Workshop (2014) were used (24). First, the removed parathyroid glands were fixed in formalin and immediately submitted for extemporaneous histological analysis. Each gland was transmitted with the precise location of the sample. Intra-operative PTH was measured after the removal of all hyperfunctional glands. A final histological report was established in compliance with the WHO Classification of Tumours of Endocrine Organs (25), in particular by describing the contours of the lesions, presence or not of a rim sign, respect or not of the trabecular architecture, and mitotic index. The Ki67 expression was not performed systematically in all study centers before 2016; for this reason, data concerning this proliferation marker could not be collected and analyzed. Histological examination was used as gold standard.

### [^18^F]Fluorocholine positron emission tomography/computed tomography

[^18^F]Fluorocholine PET/CT images were acquired using two PET/CT scanners (Discovery 710; General Electric Healthcare and Biograph 20 mCT Flow; Siemens Healthineers) between 45 and 60 min after intravenous injection of 3 Mbq/kg of [^18^F]fluorocholine. PET/CT was conducted from the upper neck to the abdomen. [^18^F]fluorocholine PET/CT images were acquired for 2 min/bed position for Discovery 710 and at a speed 0.4 mm/s for Biograph mCT Flow. PET images were reconstructed with non-contrast low-dose CT images for Biograph mCT Flow and with contrast low-dose CT images for Discovery 710. In the first center (Biograph mCT Flow), the CT parameters used were 120 kV, 120 mAs, collimation of 20 × 0.6 mm, and a pitch of 1.3, with a slice thickness of 5 mm. PET images were reconstructed with an iterative 3-dimensional method using 2 iterations, 21 subsets, and a Gaussian filter (FWHM, 5 mm). In the second center (Discovery 710) the CT parameters used were 120 kV, 120 mAs, collimation of 20 × 1.25 mm, and a pitch of 1.38, with a slice thickness of 2.5 mm. PET images were reconstructed with an iterative 3-dimensional method using 4 iterations, 24 subsets, and a Gaussian filter (FWHM, 5mm).

### Overall performance of [^18^F]fluorocholine positron emission tomography/computed tomography with visual analysis and definition of true negative

Overall [^18^F]fluorocholine PET/CT sensitivity, specificity, positive predictive value (PPV), negative predictive value (NPV), and accuracy were calculated on a per-lesion analysis and on a per-patient analysis after a visual analysis. For this analysis, the nuclear physician was not blinded to the other imaging and biological results. [^18^F]fluorocholine PET/CT was considered as true positive if a clear focal uptake was identified and if histology showed an adenoma or a hyperplasia. All true positives were histologically proved.

[^18^F]Fluorocholine PET/CT was considered as true negative if there was no clear focal uptake and (i) in case of surgery: histology did not find adenomas or hyperplasias; (ii) in case of multidisciplinary decision not to operate and to monitor the patient: there was no evolution over 1-year follow-up (no appearance of symptoms or worsening of biological abnormalities).

[^18^F]Fluorocholine PET/CT was considered as false positive if a clear focal uptake was identified and if histology did not find adenoma or hyperplasia. [^18^F]Fluorocholine PET/CT was considered as false negative if there was no clear focal uptake and histology showed an adenoma or a hyperplasia. Rate detection was calculated as the ratio of true positives divided by the number of [^18^F]fluorocholine PET/CT performed. Rate detection was measured in a per-patient analysis for subgroups of serum PTH level: <70 (7.42 pmol/L), 70–120 (7.42–12.72 pmol/L), and >120 ng/ml (12.72 pmol/L).

### Semi-quantitative analysis

Secondarily, quantitative measurements were performed by a nuclear medicine physician who was blinded to histology, laboratory results, first-line imaging, and the result of the first overall visual analysis. Images were analyzed using the syngovia software (Siemens Healthcare, Erlangen, Germany). Four criteria were measured or calculated, namely, maximum SUV_max_, liver ratio, thyroid ratio, and size ratio. The performance of each criteria was represented by the area under the receiver operating characteristic (ROC) curve (AUC) and Younden index, positive and negative likelihood ratios (LR+ and LR-), and accuracy.

For each lesion, the following parameters were measured: (i) SUV_*max bw*_ (standard uptake value maximum body weight): if the exam was considered normal, without target lesion, the mediastinal background noise was measured and taken account for the ROC curve analysis; (ii) SUV_*mean bw*_; (iii) SUV_*peak bw*_; (iv) SUV_max *liver bw*_: measurements on the liver were normalized: ROI drawn in the right liver, at the limit of segments VII and VIII, with a standardized size of 3 cm in diameter. To avoid the risk of overestimation, measurements were not performed within the acquisition limits. PET imaging studies excluding the liver from the scope of acquisition were excluded; (v) SUV_*max thyroid bw*:_ measurements taken as desired on the right or left lobe, keeping a distance from any nodule. Thyroid background was not measured in case of pathological thyroid (for instance in case of thyroidectomy or thyroiditis); (vi) size of the lesion in millimeters on the CT (not evaluated for negative [^18^F]fluorocholine PET/CT).

Composite criteria were defined: (i) the Liver Ratio which is the ratio between the SUV_max_ of the lesion on the background noise of the liver multiplied by one hundred; (ii) the Thyroid Ratio which is the ratio between the SUV_max_ of the lesion on the background noise of the thyroid multiplied by ten; (iii) the Size Ratio which is the ratio between the SUV_max_ of the lesion on the size in millimeters multiplied by ten.

### Semi-quantitative analysis of histologically proven lesions only

In order not to be influenced by the definition of true negatives that we have opted for, we performed a second analysis excluding non-operated patients. We wanted to strengthen the robustness of our results with the second analysis.

### Statistical analysis

A statistical analysis was performed using commercial software (GraphPad Prism, La Jolla, CA, United States). Receiver Operating Characteristic (ROC) curve were performed using Wilson/Brown method. An ideal criterion would have an area under the curve (AUC) of 1, and a criterion with no discrimination utility has an AUC of 0.5. A correlation test between two outcomes was carried out by Mann–Whitney test. For all the tests, a *P* value of 0.05 or less was considered statistically significant.

## Results

### Study population

**FIGURE 1:**
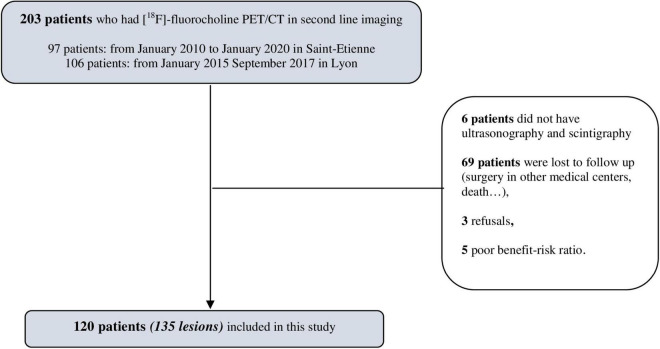
Flowchart.

The [^18^F]fluorocholine PET/CT data from 203 patients were screened. One hundred and twenty patients were finally included in the final analysis, and this corresponded to 135 lesions (Figure 1). The vast majority of patients became lost to follow-up because the examination was initially requested by peripheral hospitals where patients returned for their surgery. The baseline characteristics of the 120 patients included are presented in Table 1. All the patients had biological hyperparathyroidism and a mean serum calcium level of 2.78 ± 0.33 mmol/L and a serum PTH level of 127.19 ± 85.05 pmol/L. Ninety-six of the patients underwent surgery. After surgery, the average serum PTH level decreased from 127.19 ± 85.05 to 23.11 ± 29.79 pmol/L, or about 80%. Sixty-one (50.8%) of the patients were symptomatic.

**TABLE 1:** Population characteristics and biological results.

Population	*n* = 120
Age (years)*	64.6 ± 13.89
**Sex–no (%)**	
Female	79 (65.8)
Male	41 (34.2)
Symptomatic–no (%)	61 (50.8)
Ectopic–no (%)	17 (14.3)
Primary hyperparathyroidism–no (%)	100 (83.3)
Secondary or tertiary hyperparathyroidism–no (%)	20 (16.7)
Pre-surgery characteristics*	
Size (mm)	10.28 ± 5.43
Serum calcium (mmol/L)	2.78 ± 0.33
Serum PTH (pmol/L)	127.19 ± 85.05
[^18^F]fluorocholine (MBq)	218.27 ± 56.03
CTDI (mGy)	14.82 ± 10.31
PDL (mGy.cm)	566.52 ± 301.15
Post-surgery characteristics*	*n* = 96
Serum PTH (pmol/L)	23.11 ± 29.79
Variation** PTH (pmol/L)	92.65 ± 67.05

*Mean ± SD (range).

**Variation between pre- and post-surgery serum PTH level.

Serum calcium standards at the Lyon laboratory: 2.04–2.39 mmol/L.

Serum calcium standards at the Saint Etienne laboratory: 2.1–2.55 mmol/L.

Serum PTH level standards at the Lyon laboratory: 15–57 ng/L.

Serum PTH level standards at the Saint Etienne laboratory: 5.5–38.4 ng/L.

Serum calcium levels are corrected to albumin or protide concentration.

CTDI, computed tomography dose index; DLP, dose length product; PTH, parathyroid hormone; SUV_max_, maximum standardized uptake value.

### Per lesions analysis

On a per-lesion analysis the test for overall sensitivity, specificity, PPV, NPV, and accuracy was 99.04, 93.33, 97.17, 96.55, and 97.78% (Table 2). The histological results of 111 lesions were collected. Seventeen (14.3%) hyperfunctional glands were ectopic. One hundred and four lesions (104/135) were considered as [^18^F]fluorocholine PET/CT true positives, corresponding to 93 adenomas and 11 hyperplasias that were histologically proven. There was no parathyroid carcinoma in this cohort.

**TABLE 2:** Sensitivity, specificity, negative predictive value (NPV), positive predictive value (PPV), and accuracy of [^18^F]fluorocholine positron emission tomography/computed tomography (PET/CT) in a per-patient analysis (*N* = 120) and in a per-lesion analysis (*N* = 135).

	Per patient	95% CI	Per lesion	95% CI
Sensitivity	98.89	93.97–99.80	99.04	94.75–99.83
Specificity	93.33	78.68–98.15	93.33	78.68–98.15
Negative predictive value (NPV)	96.55	82.82–99.39	96.55	82.82–99.39
Positive predictive value (PPV)	97.80	92.34–99.40	98.11	93.38–99.48
Accuracy	97.50	93.67–99.24	97.78	92.91–99.15

Twenty-eight lesions (28/135) were considered as true negatives: four lesions had negative [^18^F]fluorocholine PET/CT but surgically treated because of histological confirmation of differential diagnosis (lymph nodes, metastases, or normal parathyroid); twenty-four lesions had negative [^18^F]fluorocholine PET/CT, corresponding to patients without clinical symptoms and without evolution during 1 year of follow-up after pluridisciplinary surveillance decision.

Only one lesion (1/135) was a false negative. This lesion concerned a symptomatological patient with doubtful ultrasonography but had a parathyroid adenoma after surgery on the right upper gland.

Two false positives lesions (2/135) were benign lymph nodes.

### Per patient analysis

On a per-patient analysis, the test for overall sensitivity, specificity, PPV, NPV, and accuracy was 98, 93.33, 96.55, 97.8, and 97.5% (Table 2). Eighty-nine patients (89/120) were considered as [^18^F]fluorocholine PET/CT true positives, corresponding to 93 adenomas and 11 hyperplasias that were histologically proven. Twenty-eight patients (28/120) were considered as true negatives.

Only one patient (1/120) had a false negative [^18^F]fluorocholine PET/CT, symptoms, and doubtful ultrasonography but had a parathyroid adenoma after surgery on the right upper gland. Two false positives patients (2/120) had positive [^18^F]fluorocholine PET/CT and benign lymph nodes.

### Semi-quantitative analysis

**FIGURE 2:**
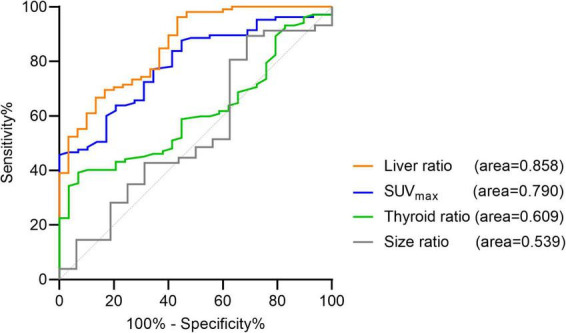
Receiver operating characteristic (ROC) curves for the quantitative criteria. Liver ratio: AUC = 0.858; 95% CI (0.785–0.929), *p* < 0.0001. SUV_max_: AUC = 0.79; 95% CI (0.708–0.872), *p* < 0.0001. Thyroid ratio: AUC = 0.609; 95% CI (0.506–0.712), *p* = 0.0738. Size ratio: AUC = 0.539; 95% CI (0.38–0.698), *p* = 0.6126.

The AUC representing SUV_max_ and liver ratio was significantly increased. It was 0.858 [95% IC (0.785–0.929), *p* < 0.0001] for liver ratio and 0.79 [95% IC (0.708–0.872), *p* < 0.0001] for SUV_max_. The AUC for thyroid ratio and size ratio did not increase (Figure 2). For twenty-eight negative [^18^F]fluorocholine PET/CT, size could not be measured. For three patients, thyroid background could not be determined because of thyroidectomy or thyroiditis.

The optimal cut-off values represented by the maximum Youden index were for SUV_max_ > 4.12 [Youden index = 0.457, Se = 45.71% (36.51–55.23), Sp = 100% (88.3–100), LR +> 100, LR -= 0.543, accuracy = 0.577] and for liver ratio > 27.4 [Youden index = 0.533, Se = 66.67% (57.2–74.95), Sp = 86.67% (70.32–94.65), LR += 5.002, LR -= 0.38, accuracy = 0.711]. The Youden index for unblind and global visual interpretation of [^18^F]fluorocholine PET/CT in our study is 0.92 vs. about 0.5 for SUV_max_ and liver ratio used alone. For SUV_max_ > 4.12 or liver ratio > 38.1, specificity was 100% [88.3–100], but sensitivity was less than 50%.

### Semi-quantitative analysis of histologically proven lesions only

The AUC representing SUV_max_ and liver ratio remained significantly increased but with less statistical strength. AUC was 0.833 [95% CI (0.69–0.976), *p* = 0.006] for liver ratio and 0.824 [95% CI (0.682–0.965), *p* = 0.008] for SUV_max_. The AUC for thyroid ratio and size ratio did not increase. The optimal cut-off values represented by the maximum Youden index were for SUV_max_ > 4.03 [Youden index = 0.4667, Sp = 100.00% (60.97–100), Se = 46.67% (37.41–56.16)] and for liver ratio > 32.15 [Youden index = 0.524, Sp = 100% (60.97–100), Se = 55.24% (45.71–64.4)].

### Subgroups of lesions

**FIGURE 3:**
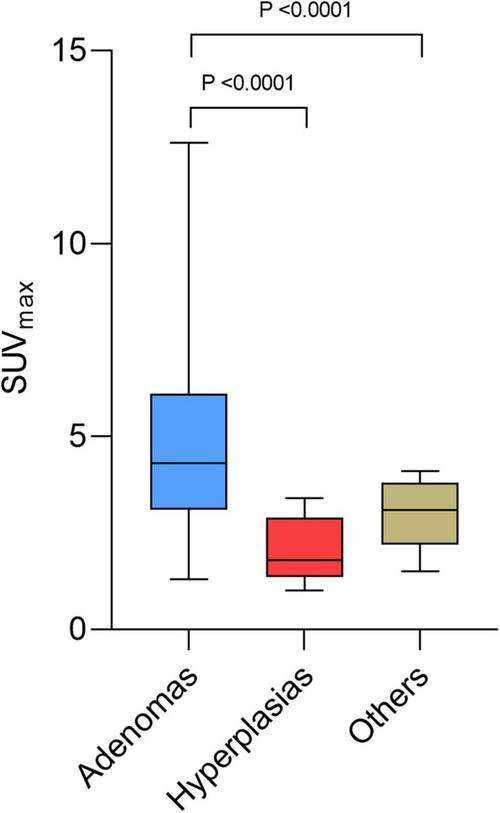
Box plots of maximum standardized uptake value (SUV_max_) for adenoma, hyperplasia, and other diagnosis.

**FIGURE 4:**
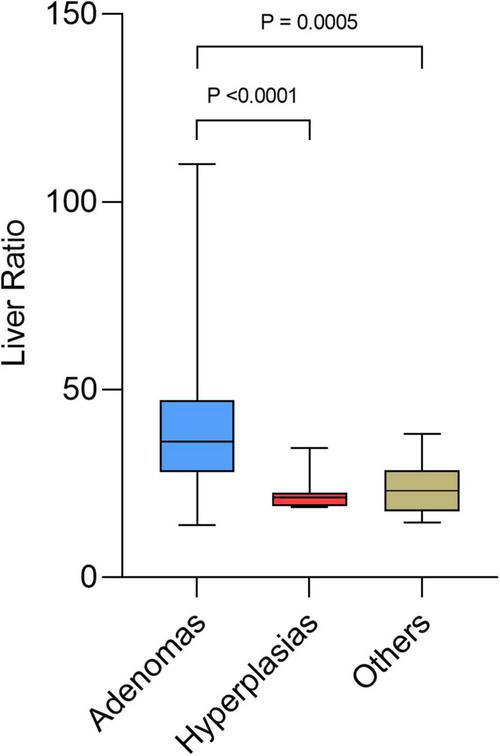
Box plots of liver ratio for adenoma, hyperplasia, and other diagnosis.

Table 3 shows the significant differences between the subgroups of lesions: adenomas, hyperplasias, and other diagnoses only proven histologically. Differential diagnoses were normal parathyroids, one lymph node metastasis from a small cell lung cancer (26), and benign lymph nodes. Hyperfunctional glands had a higher SUV_max_ (*p* = 0.0085) and a higher liver ratio (*p* = 0.0002) than differential diagnosis. SUV_max_ and liver ratio were significantly higher for adenomas than for hyperplasias (*p* < 0.0001) (Figures 3, 4).

**TABLE 3:** Difference in quantitative criteria among adenoma, hyperplasia, and other diagnosis.

				*P*-value	
				
	Adenomas	Hyperplasias	Others*	Between adenomas and hyperplasias	Between adenomas and other diagnosis
SUV_max_	4.89 ± 2.41	2.11 ± 0.85	2.98 ± 0.85	<0.0001	0.0005
Liver ratio	40.02 ± 19.12	21.79 ± 4.11	23.40 ± 6.88	<0.0001	<0.0001

P value of Mann–Whitney test.

Mean ± SD (range).

*Lymph nodes, metastases, or histologically proven normal parathyroids.

The mean SUV_max_ of adenomas was 4.89 ± 2.41, while that of hyperplasias was 2.11 ± 0.85, and that of other diagnosis was 2.46 ± 1.05. Mean thyroid ratio and mean size ratio were not significantly different. When the SUV_max_ > 4.12 or the liver ratio > 38.1, 100% of the lesions were histologically proven adenomas. When the liver ratio is greater than 32.95, 96.65% of lesions were histologically proven adenomas.

### Subgroups of serum parathyroid hormone level

**FIGURE 5:**
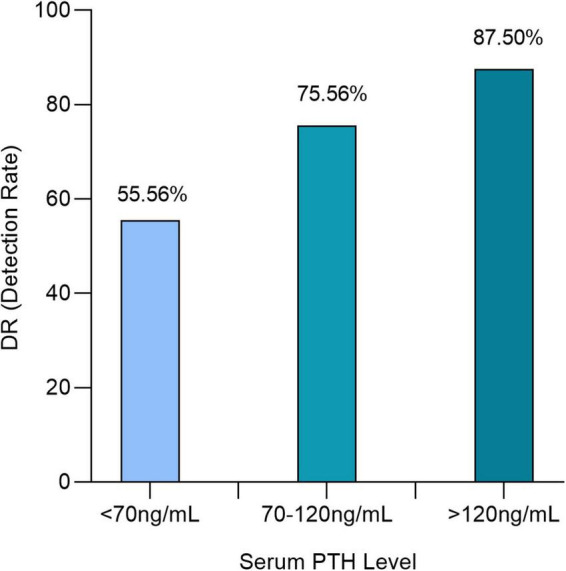
Detection rate for [^18^F]fluorocholine positron emission tomography/computed tomography (PET/CT) for each subgroups of serum parathyroid hormone (PTH) level: <70, 70–120, and >120 ng/ml.

The positivity of [^18^F]fluorocholine PET/CT was correlated with the serum level PTH (*P* = 0.0082). Detection rates were 55.56 (15/27), 75.56 (34/45), and 87.5% (42/48) for serum PTH levels less than 70 (7.42 pmol/L), 70–120 (7.42–12.72 pmol/L), and greater than 120 ng/ml (12.72 pmol/L) (Figure 5). In a subgroup analysis, we were able to confirm this difference in each center regardless of laboratory PTH standards.

## Discussion

The results of our study confirm the excellent diagnostic performance of [^18^F]fluorocholine PET/CT for localization of hyperfunctional parathyroid glands in second-line imaging compared to a histological gold standard. Sensitivity, specificity, NPV, PPV, and accuracy are consistent with those reported in the literature (10, 11). One of the strengths of our study is histological evidence in 82% of the cases.

We carried out a semi-quantitative analysis preceded by a visual analysis. This allowed for us to measure the performance of quantitative measures compared to the overall performance of the exam. It is important to highlight that quantitative criteria are not sufficient to locate pathological parathyroid glands. When interpreting nuclear imaging, a physician relies on a bundle of arguments: clinical symptoms, biological results, and results of other imaging modalities. Our study highlights two quantitative tools useful for [^18^F]fluorocholine PET/CT interpretation: SUV_max_ and liver ratio. These quantitative parameters have lower overall performance but higher specificity than an overall visual analysis in identifying an adenoma. Taking into account the liver background seems to be a good way to standardize SUV measurements to each individual (27). Other quantitative parameters such as thyroid ratio and size ratio do not appear useful in increasing the performance of the imaging.

The major interest of the quantitative criteria is their ability to distinguish an adenoma from a hyperplasia. Beyond certain threshold values of SUV_max_ and liver ratio (SUV_max_ > 4.12 or liver ratio > 38.1 in our study), all lesions are adenomas. Treglia et al. and Piccardo et al. (10, 22) also found higher SUV_max_ for adenomas than for hyperplasias. Identifying the difference between a hyperplasia and an adenoma can be a major issue for surgeons in order to choose between MIP and bilateral cervical exploration, especially in case of multiple targets (12, 13, 28). The indicated SUV values in the final report could allow for better management of surgical procedures and better communication among physicians, surgeons, and clinicians with less subjective identification of lesions.

Our last finding was the direct influence of the serum PTH level on the results of the [^18^F]fluorocholine PET/CT, with significant decrease performance for serum PTH level < 70 ng/ml. Nevertheless, even with serum PTH level at the limit of normal, [^18^F]fluorocholine PET/CT localized a lesion in 55.56% of cases of hyperparathyroidism. Araz et al. (21), found that SUV_max_ > 4.4 was correlated with high serum PTH level. Alharbi et al. (29) found that uptake adenoma is correlated with PTH serum level. Bossert et al. (17) found that PET/CT is efficient for patients with normocalcemic hyperparathyroidism. To the best of our knowledge, no other study reported on detection rates depending on serum PTH level. This approach has been performed on prostate cancers for PSA level and [^18^F]fluorocholine PET/CT or PSMA-targeted PET imaging. This could also be studied for [^99m^Tc]Tc-Sestamibi scintigraphy, 4D-CT, and MRI to know if [^18^F]fluorocholine PET/CT represented any diagnostic improvement over other imaging modalities when the level of PTH is low. In the literature, [^99m^Tc]Tc-Sestamibi Scintigraphy sensitivity appears to be decreased sharply when serum PTH level is low (30). Moreover, it has been shown that serum PTH level was correlated with the size of parathyroid lesions (28, 31). It cannot be determined by this study whether the decrease in detection rate when serum PTH level is low is due to decrease in the size of the lesions.

Our study has some limitations. First, our population does not represent the general population but patients who have already had inconclusive first-line imaging, corresponding to the current indication for [^18^F]fluorocholine PET/CT in Europe (20). The question of replacing the first-line imaging with systematic [^18^F]fluorocholine PET/CT is not one of the objectives of our study and depends on large prospective multicenter studies and cost-effectiveness analyses. Second, the definition of true negative patients in the absence of surgery could be discussed. As Treglia et al. (10) pointed out, true negatives are often not clearly defined in literature (20), and there is no consensus on this particular point. A year of follow-up without evolution is not a formal proof of absence of adenoma or hyperplasia. To confirm our results and increase their robustness, a second analysis excluding patients without surgery was performed and confirmed our findings but with less statistical strength. The mean values of the quantitative criteria were calculated only with histologically proven lesions. For patients considered true negatives who did not have surgery, beyond 1 year, it might be interesting to repeat [^18^F]fluorocholine PET/CT.

Moreover, as we condition the performance of our test compared to histology, some patients screened for the study were lost to follow-up and excluded from the analysis, which could generate attrition bias. The large number of patients lost to follow-up may be partly explained by the fact that the nuclear medicine departments in the study are regional centers that perform scintigraphy and PET/CT for many peripheral hospitals. Patients not operated in one the inclusion centers were excluded to homogenize surgical decision and follow-up. We wanted this homogenization to strengthen the definition of true negatives that we have chosen to adopt. In addition, the significant difference in number of lesions, 93 adenomas vs. 11 hyperplasias, decreases the statistical comparability of the subgroups, but this is the usual distribution in the epidemiological data (32). Finally, the use in one center and not in the other of contrast-enhanced CT attenuation correction can result in a variation in SUV value (33).

Despite these considerations, our data propose the first evaluation of [^18^F]fluorocholine PET/CT quantitative parameters’ performance in a large cohort of patients with adenoma or hyperplasia.

## Conclusion

Semi-quantitative parameters (SUV_max_ and liver ratio) should be considered as additional tools in interpretation of [^18^F]fluorocholine PET/CT. These parameters have an interest to differentiate adenomas from hyperplasias and differential diagnosis. These quantitative parameters have lower overall performance but higher specificity than an overall visual analysis in identifying an adenoma. Above certain threshold values, all lesions are adenomas.

[^18^F]Fluorocholine PET/CT is confirmed to have an excellent detection efficacy for hyperfunctioning parathyroid glands in second-line imaging. For serum PTH levels < 70 ng/ml, detection rate is strongly decreased, but it managed to identify a lesion in more than half of the cases.

## Data availability statement

The original contributions presented in this study are included in the article/Supplementary Material, further inquiries can be directed to the corresponding author.

## Ethics statement

The studies involving human participants were reviewed and approved by French Ethics Committee for Research in Nuclear Medicine (Record Number: CEMEN2021-06). Written informed consent for participation was not required for this study in accordance with the national legislation and the institutional requirements.

## Author contributions

NP: principal investigator at Saint-Etienne Hospital, nuclear physician having interpreted 18F-Choline PET/CT and practice semi quantitative analysis, and major contributions to drafting of the manuscript. NJ-F: investigator and project holder, management of the statistical analysis in contact with the public health department of the University Hospital of Saint-Étienne, nuclear physician having interpreted 18F-Choline PET/CT and practice semi quantitative analysis, and major contributions to drafting of the manuscript. IM: principal investigator at Lyon Hospital and nuclear physician having interpreted 18F-Choline PET/CT. P-BB: nuclear physician having interpreted 18F-Choline PET/CT and major contributions to drafting of the manuscript. J-MP: privileged interlocutor for parathyroid surgery and participation in multidisciplinary decisions. NG and CM: patient referral clinicians and participation in multidisciplinary decisions. VH: management of the statistical analysis and nuclear physician having interpreted 18F-Choline PET/CT. All authors contributed to the article and approved the submitted version.
