# Supplementary figures and images for: Performance of quantitative measurements in [18F]fluorocholine positron emission tomography/computed tomography for parathyroid imaging (P2TH study)
Source: Front Med (Lausanne). 2022 Aug 2;9:956580. doi: 10.3389/fmed.2022.956580 (PMC9380568; doi:10.3389/fmed.2022.956580)

## ROC Curves v2

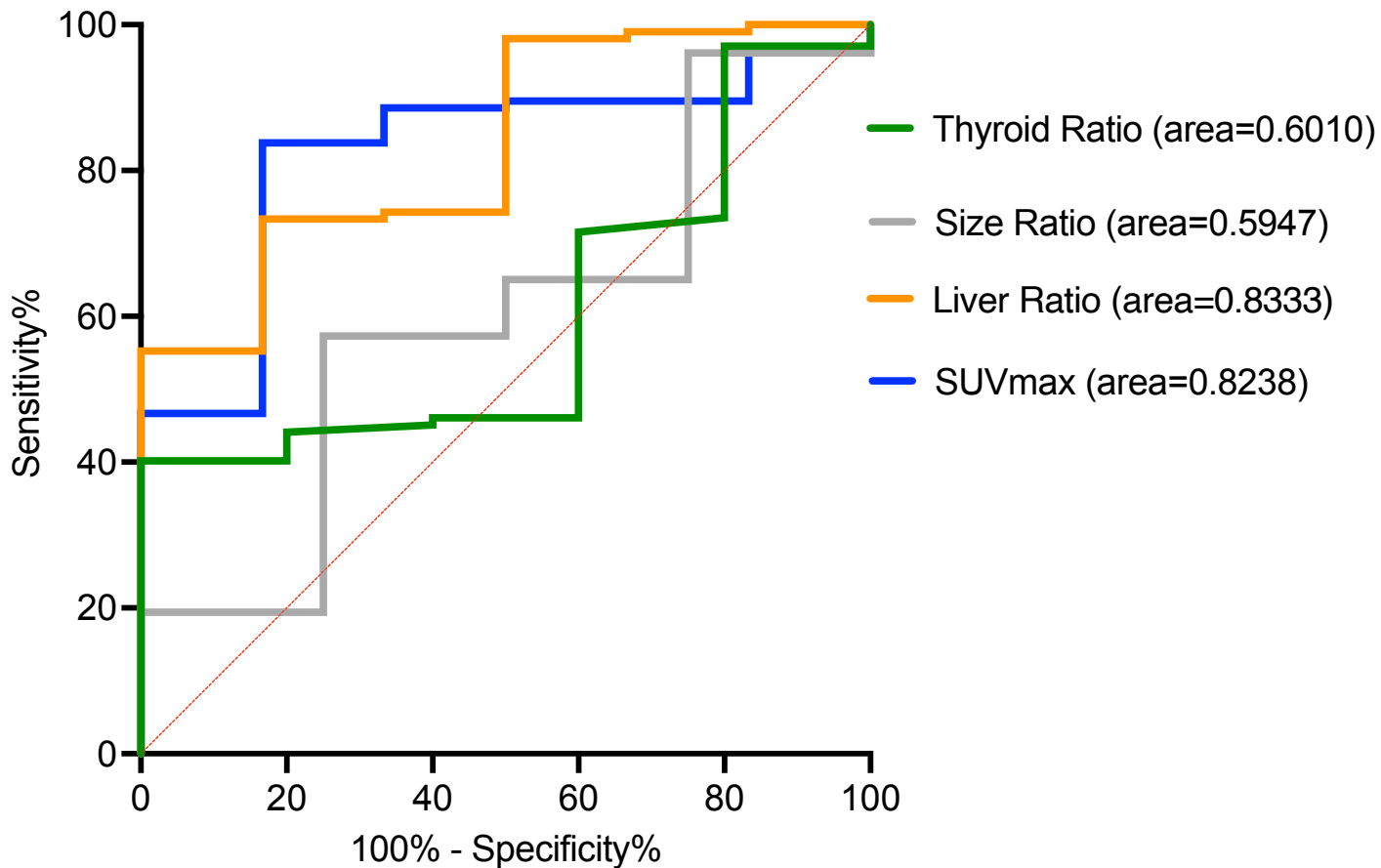

Supplement: Supplementary file 1 [file Image_1.pdf]
